# New synonymies and combinations in *Argyrostrotis* Hübner (Lepidoptera, Erebidae, Erebinae, Poaphilini)

**DOI:** 10.3897/zookeys.149.2347

**Published:** 2011-11-24

**Authors:** J. Bolling Sullivan, J. Donald Lafontaine

**Affiliations:** 1 200 Craven St., Beaufort, North Carolina 28516 USA; 2 Canadian National Collection of Insects, Arachnids, and Nematodes, Biodiversity Program, Agriculture and Agri-Food Canada, K.W. Neatby Bldg., 960 Carling Ave., Ottawa, Ontario, Canada K1A 0C6

**Keywords:** *Argyrostrotis*, *Argyrosticta eurysaces*, *Ptichodis surrufula*, *Heterochroma quadrata*, eastern North America

## Abstract

After examining the type specimens of species in the eastern North American genus *Argyrostrotis* the number of known species in the genus is reduced from 10 to six through synonymy. A key to species is included along with illustrations of the adults and genitalia of each species. Three Neotropical species currently included in *Argyrostrotis* (*Argyrostrotis eurysaces* Schaus, 1914; *Argyrostrotis quadrata* Dognin, 1910; and *Celiptera surrufula* Dyar, 1913) are transferred to other genera as *Argyrosticta eurysaces* (Schaus, 1914), **comb. n.** [Noctuidae: Bagisarinae], *Heterochroma quadrata* (Dognin, 1910), **comb. n.** [Noctuidae: Amphipyrinae], and *Ptichodis surrufula* (Dyar, 1913), **comb. n.** [Erebidae: Erebinae: Euclidiini].

## Introduction

Currently, there are 10 species of *Argyrostrotis* Hübner listed by Lafontaine and Schmidt (2010). An additional three species listed by Poole (1989) from Mexico and South America are transferred to other genera, thereby restricting the geographic range of the genus to eastern and central North America. Examination of the type specimens, and the published illustrations associated with the original descriptions where the types have been lost or destroyed, shows that four of the names recognized as valid species should be placed in synonymy with *Argyrostrotis flavistriaria* (Guenée). A key to species and adults and genitalia of each species are illustrated in order to facilitate identification.

## Materials and methods

### Repository abbreviations

Specimens were examined from the following collections:

AMNH American Museum of Natural History, New York, New York, USA


BMNH The Natural History Museum (statutorily, British Museum (Natural History)), London, UK


CNC Canadian National Collection of Insects, Arachnids, and Nematodes, Ottawa, Ontario, Canada


JBS Personal collection of J. Bolling Sullivan, Beaufort, North Carolina, USA


MNHN Muséum national d’histoire naturelle, Paris, France


USNM National Museum of Natural History (formerly, United States National Museum), Washington, District of Columbia, USA


### Dissecting methods and terminology

Dissection of genitalia and terms for genital structures and wing markings follow Lafontaine (2004).

### Key to species of *Argyrostrotis*

**Table:** 

1	Forewing with postmedial line straight or evenly curved, usually prominent	2
–	Forewing with postmedial line dentate, usually obscure	5
2	Forewing with postmedial line appearing to extend to apex and usually highlighted by yellow line or spots	*Argyrostrotis flavistriaria*
–	Forewing with postmedial line subapical and without yellow shading	3
3	Forewing with postmedial line curved toward wing base at costa; basal, medial, and terminal areas may be extensively dusted with white scales	*Argyrostrotis sylvarum*
–	Forewing with postmedial line straight; ground color brown	4
4	Postmedial line complete	*Argyrostrotis quadrifilaris*
–	Postmedial line incomplete	*Argyrostrotis anilis*
5	Forewing length 15–17mm; fringe with white scaling	*Argyrostrotis erasa*
–	Forewing length 10–13 mm; fringe rarely with white scaling and if so, scaling minute	*Argyrostrotis deleta*

## Systematics

### Argyrostrotis flavistriaria

(Hübner, [1831])

http://species-id.net/wiki/Argyrostrotis_flavistriaria

**Figures 1–10.**
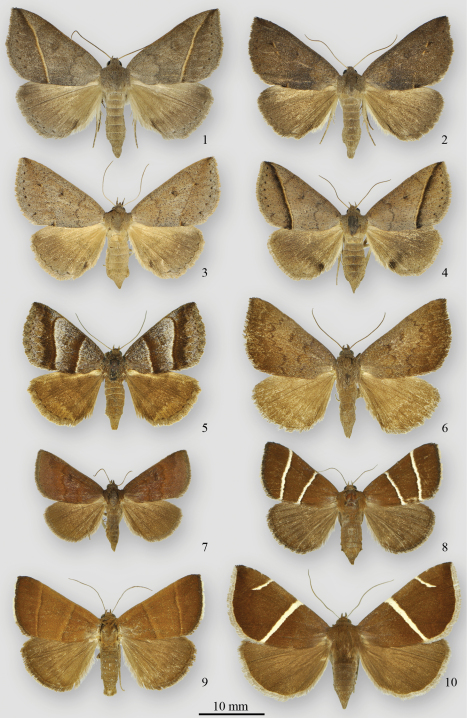
*Argyrostrotis* adults **1–4** *Argyrostrotis flavistriaria* **5** *Argyrostrotis sylvarum* **6** *Argyrostrotis erasa* **7** *Argyrostrotis deleta* **8, 9** *Argyrostrotis quadrifilaris* **10** *Argyrostrotis anilis*.

Figs 1–4 11–15 20 26

Crochiphora flavistriaria Hübner, [1831]: 35, pl. [96], figs 555, 556.
Poaphila herbicola Guenée, 1852: 301, syn. n.
Poaphila contempta Guenée, 1852: 302, syn. n.
Poaphila perplexa Guenée, 1852: 302.
Poaphila perspicua Walker, 1858: 1477.
Mocis? diffundens Walker, 1858: 1491, syn. n.
Phurys glans Grote, 1876b: 416.
Phurys carolina Smith, 1905: 68, syn. n.

#### Type material.

**Figures 11–19.**
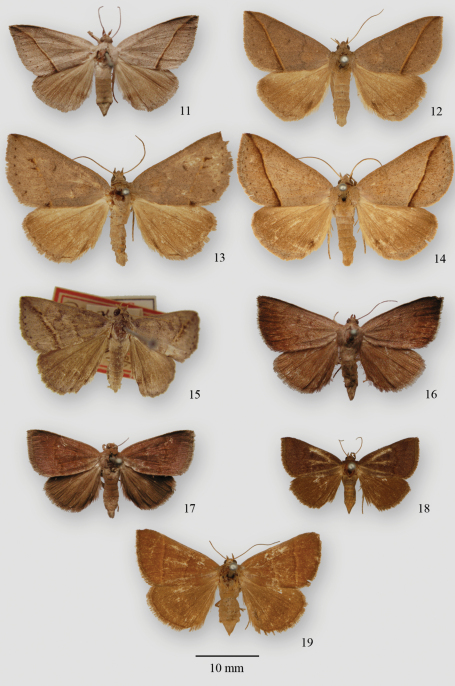
Type material of *Argyrostrotis* **11** *Poaphila perplexa* lectotype, MNHN **12** *Poaphila perspicua* holotype, BMNH **13** *Mocis? diffundens* holotype, BMNH **14** *Phurys glans* syntype, BMNH **15** *Phurys carolina* lectotype, AMNH **16** *Poaphila erasa* lectotype, MNHN **17** *Poaphila deleta* lectotype, MNHN **18** *Poaphila placata* syntype, BMNH **19** *Poaphila obsoleta* syntype, BMNH.

 The type material of *Crochiphora flavistriaria* is lost, but the illustrations (Hübner, 1831, pl. [96], figs 555, 556) are diagnostic and represent the form shown in Fig. 1. The type specimens of *Poaphila herbicola* and *Poaphila contempta* are lost, but the original descriptions are diagnostic and represent the forms shown in Figs 2 and 3 respectively. The female lectotype of *Poaphila perplexa* in the MNHN labelled “Javana [Savannah] Georgia/ *perplexa*/ Type/ Museum Paris/ *Poaphila perplexa* Gn. Vol. 7, 1852, p. 302, n=1755” is shown in Fig. 11. The male holotype of *Poaphila perspicua* in the BMNH labelled “Type/ *Argyrostrotis perspicua*.” is shown in Fig. 12 and represents the same form as the original illustration of *Crochiphora flavistriaria*. The male holotype of *Mocis? diffundens* in the BMNH labelled “Type/ 8. *Mocis? diffundens*.” is shown in Fig. 13. A male syntype of *Phurys glans* in the BMNH labelled “Type/ Grote Coll. 82-54./ 3129/ *Phurys glans* Grote type” is shown in Fig. 14. The male lectotype of *Phurys carolina* in the AMNH labelled “*Phurys carolina* Smith % type/ Nth Car., August”/ Coll. J.B. Smith/ Lectotype by E.L. Todd” is shown in Fig. 15.

#### Distribution.

 North Carolina south to Florida and Texas.

### Argyrostrotis sylvarum

(Guenée, 1852)

http://species-id.net/wiki/Argyrostrotis_sylvarum

Figs 5 21 27

Poaphila sylvarum Guenée, 1852: 300, pl 23, fig 2.

#### Type material.

 The type material of *Poaphila sylvarum* is lost but the original description and associated illustration are diagnostic.

#### Distribution.

 Virginia south to Florida and Texas.

### Argyrostrotis erasa

(Guenée, 1852)

http://species-id.net/wiki/Argyrostrotis_erasa

Figs 6 16 22 28

Poaphila erasa Guenée, 1852: 301.

#### Type material.

 The female lectotype of *Poaphila erasa* labelled “Javana [Savannah] Georgia/ Poaphila erasa Gn./ Type/ Poaphila erasa Gn. Vol. 7, 1852 p. 301, n=1751” in the MNHN is shown in Fig. 16 [forewing length 17 mm].

#### Distribution.

 North Carolina south to Florida and Texas.

### Argyrostrotis deleta

(Guenée, 1852)

http://species-id.net/wiki/Argyrostrotis_deleta

Figs 7 17, 18 23 29

Poaphila deleta Guenée, 1852: 300.
Poaphila placata Grote, 1878: 184.

#### Type material.

 The male lectotype of *Poaphila deleta* labelled “Javana [Savannah] Georgia/ *Poaphila deleta*/ Type/ *Poaphila deleta* Gn. Vol. 7, 1852, p. 300, n=1748” in the MNHN is shown in Fig. 17 [forewing length 13 mm]. A female syntype of *Poaphila placata* in the BMNH labelled “Georgia, Grote Coll. 82–54./ *Poaphila placata* Grote Type” is shown in Fig. 18.

#### Distribution.

 Virginia south to Florida and Texas.

### Argyrostrotis quadrifilaris

(Hübner, [1831])

http://species-id.net/wiki/Argyrostrotis_quadrifilaris

Figs 8, 9 19 24 30

Agronomia quadrifilaris Hübner, [1831]: 37, pl. [98], figs 569, 570.
Poaphila obsoleta Grote, 1876a: 42.

#### Type material.

 The type specimen of *Agronomia quadrifilaris* is lost, but the illustrations (Hübner, 1831, pl. [98], figs 569, 570) are diagnostic and represent the form shown in Fig. 8. A female syntype of *Poaphila obsoleta* in the BMNH labelled “Enterprise, Florida, 12.V. Grote Coll. 82-54./ *Poaphila obsoleta* Grote Type” is shown in Fig. 19.

#### Distribution.

 New York and New Hampshire south to Florida and Texas.

### Argyrostrotis anilis

(Drury, 1773)

http://species-id.net/wiki/Argyrostrotis_anilis

Fig. 10 25 31

Phalaena anilis Drury, 1773: 21, pl. 12, fig. 21.
Agronomia sequistriaris Hübner, [1831]: 10, pl. [73], figs 419, 420.

#### Type material.

 The type specimen of *Phalaena anilis* is lost, but the illustration in Drury (1773) is diagnostic, as are those of *Agronomia sequistriaris* in Hübner, [1831].

#### Distribution.

 Southern Canada (Quebec to Saskatchewan) south to Florida and Texas.

##### Excluded species

Three species included in *Argyrostrotis* by Poole (1989) are hereby excluded from the genus.

*Argyrostrotis eurysaces* Schaus, 1914 is hereby transferred to the genus *Argyrosticta* Hübner, [1821] as *Argyrosticta eurysaces* (Schaus, 1914), comb. n. [Noctuidae: Bagisarinae]. The two genera are not closely related and the association was more likely an error in confusing the two similar generic names by Schaus than an intended placement in *Argyrostrotis*.

*Argyrostrotis quadrata* Dognin, 1910 is hereby transferred to the genus *Heterochroma* Guenée as *Heterochroma quadrata* (Dognin, 1910), comb. n. [Noctuidae: Amphipyrinae].

*Celiptera surrufula* Dyar, 1913, included in *Argyrostrotis* by Hampson (1913) and maintained there by Poole (1989), is hereby transferred to the genus *Ptichodis* Hübner, 1818 as *Ptichodis surrufula* (Dyar, 1913), comb. n. [Erebidae: Erebinae: Euclidiini].

**Figures 20–25.**
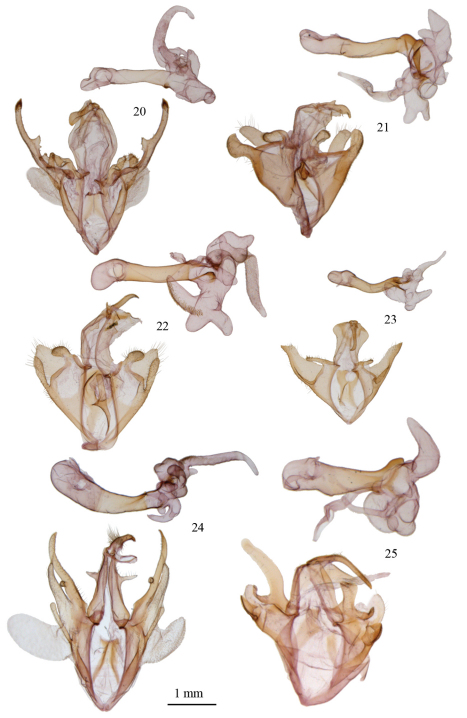
Male genitalia of *Argyrostrotis* **20** *Argyrostrotis flavistriaria* **21** *Argyrostrotis sylvarum* **22** *Argyrostrotis erasa* **23** *Argyrostrotis deleta* **24** *Argyrostrotis quadrifilaris* **25** *Argyrostrotis anilis*.

**Figures 26–31.**
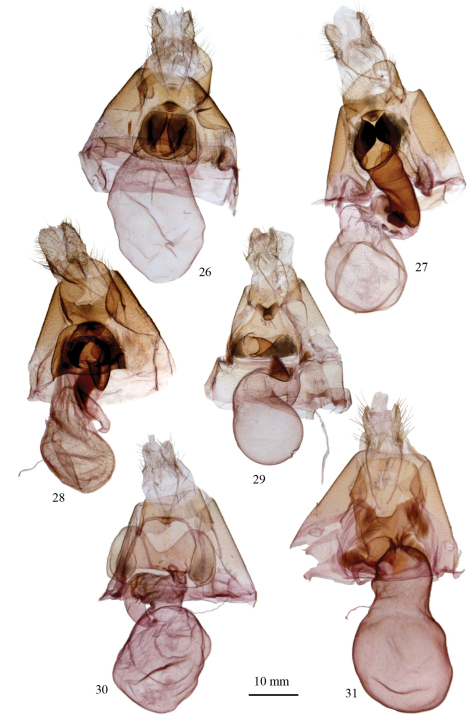
Female genitalia of *Argyrostrotis*. **26** *Argyrostrotis flavistriaria* **27** *Argyrostrotis sylvarum* **28** *Argyrostrotis erasa* **29** *Argyrostrotis deleta* **30** *Argyrostrotis quadrifilaris* **31** *Argyrostrotis anilis*.

## Supplementary Material

XML Treatment for Argyrostrotis flavistriaria

XML Treatment for Argyrostrotis sylvarum

XML Treatment for Argyrostrotis erasa

XML Treatment for Argyrostrotis deleta

XML Treatment for Argyrostrotis quadrifilaris

XML Treatment for Argyrostrotis anilis
